# Supplementary material for: Rapid turnover of life-cycle-related genes in the brown algae
Source: Genome Biol. 2019 Feb 14;20:35. doi: 10.1186/s13059-019-1630-6 (PMC6374913; doi:10.1186/s13059-019-1630-6)
Supplement: Supplementary file 3 — Protein alignments, corresponding Gblocks html file and CDS sequences for the lineage conserved gametophyte-biased genes. (ZIP 2109 kb) [file 13059_2019_1630_MOESM3_ESM.zip › Con_GA_alignments/Ec-11_004410.aa_ali.fasta-gb.htm]

Ec-11\_004410.aa.fasta.trans.aa\_ali.fasta


## Gblocks 0.91b Results

Processed file: **Ec-11\_004410.aa.fasta.trans.aa\_ali.fasta**  
Number of sequences: **2**  
Alignment assumed to be: **Protein**  
New number of positions: **309** (selected positions are underlined in blue)

```
                         10        20        30        40        50        60
                 =========+=========+=========+=========+=========+=========+
Ec-11_004410     MAASLSSLGPPHAGPGSTAPNIGFTVAATHNRRGVINKEPLSGNTLCHKNNVERKQAIHE
MSTRG.26963.1    ------------------------------------------------------------
                                                                             


                         70        80        90       100       110       120
                 =========+=========+=========+=========+=========+=========+
Ec-11_004410     NRLASVKASLSLSTPLRMEFMDKRLSKNFHARRHHELVVLQNRKILRALEEIHSRSSTVA
MSTRG.26963.1    ------------------------------------------------------------
                                                                             


                        130       140       150       160       170       180
                 =========+=========+=========+=========+=========+=========+
Ec-11_004410     AVGATNPAAEGGSMVVPASSDGERLGSSSAPVSWKKSKDLPSASATNAGESSSSGKKLNS
MSTRG.26963.1    ------------------------------------------------------------
                                                                             


                        190       200       210       220       230       240
                 =========+=========+=========+=========+=========+=========+
Ec-11_004410     LLARRRNVKAALVARENQKMMERITRSKSLYSKEKMHQHRVCHKRILRLRKTDYTAGHLL
MSTRG.26963.1    ------------------------------------------------------------
                                                                             


                        250       260       270       280       290       300
                 =========+=========+=========+=========+=========+=========+
Ec-11_004410     AAATASRHWRGGDSRDEEGRHRHRRRNRHSQRNPRVSGGSKEYANATSAPPPVARGTSYS
MSTRG.26963.1    ------------------------------------------------------------
                                                                             


                        310       320       330       340       350       360
                 =========+=========+=========+=========+=========+=========+
Ec-11_004410     PTRGKHSRSVHRGDGGGLGDRGTKSAPRLPSLLDVEDIYGGGSILGSPNLEQCLQAAKMS
MSTRG.26963.1    ------------------------------------------------------------
                                                                             


                        370       380       390       400       410       420
                 =========+=========+=========+=========+=========+=========+
Ec-11_004410     ARSGGGGGGGGEDGSAAGGSGSRPSSRNSTAGSGNHDDDGSRPRPFTDGGVGSRVGSRHH
MSTRG.26963.1    ------------------------------------------------------------
                                                                             


                        430       440       450       460       470       480
                 =========+=========+=========+=========+=========+=========+
Ec-11_004410     RKSFRQRGGKDTAGVGGSSTGGMRGTDASLDALFHHSESLGSSVEDSSGGVRRGRYGNQR
MSTRG.26963.1    ------------------------------------------------------------
                                                                             


                        490       500       510       520       530       540
                 =========+=========+=========+=========+=========+=========+
Ec-11_004410     GRGGGGSRGDKSSGSLASSGKSADDTELDKIYNPLAFLDETQGVRVLIQATKPLEVSSHP
MSTRG.26963.1    ------------------------------------------------------------
                                                                             


                        550       560       570       580       590       600
                 =========+=========+=========+=========+=========+=========+
Ec-11_004410     GETWICFIRIMVQDPFDDQLDVRIIHDREEICQRLLSLEEANAICEHAQATSSGGGGMTP
MSTRG.26963.1    ------------------------------------------------------------
                                                                             


                        610       620       630       640       650       660
                 =========+=========+=========+=========+=========+=========+
Ec-11_004410     GDRPSLFAPRAAAAAPKRSLPLPGSASSSAAAAVPPSPTMAANPGGGGGGSGGGGGGGGG
MSTRG.26963.1    ---------------------CPAGAVADAHAVVG---GEAIAP----------------
                                       ############                          


                        670       680       690       700       710       720
                 =========+=========+=========+=========+=========+=========+
Ec-11_004410     DTAADAQRAEGVAEDDGGGGGGESGGGPQTRPSAGLKRYSARDGNLSELLSALFKEADVE
MSTRG.26963.1    ------------------GGVEVDPGISNTQVSGGLERPSKGEGSLNEFLTTLFKEADTE
                                   ##########################################


                        730       740       750       760       770       780
                 =========+=========+=========+=========+=========+=========+
Ec-11_004410     EKGFLTPEQLTSIMQNAELGLTGPELHLVIAEADDNADGTIDYYEFIPLAYNLVDAFRAR
MSTRG.26963.1    QKGFLTPEQLTSIMQHAELGLTGPELHLVIAEADDNADGTIDYFEFIPLAYNLVNAFRAR
                 ############################################################


                        790       800       810       820       830       840
                 =========+=========+=========+=========+=========+=========+
Ec-11_004410     RRACEEMDEANLDLDQQAIHALYDAEFDRLVRQLKTLCKSHDPNGTGVLPRAEFKQCISA
MSTRG.26963.1    RRACEEMDEANFELDQQAIHALYDAEFDRLVRQLKSLCKSHDPDGTGTLPRAEFKQCVSG
                 ############################################################


                        850       860       870       880       890       900
                 =========+=========+=========+=========+=========+=========+
Ec-11_004410     DRCGGLTKIESKLLLNLLPKDSHGNVVYRKLGAGLEQVRFTTLRNTVAEASASDTQKYLM
MSTRG.26963.1    DRCGGLTNIESKLLLNLLPKDSRGNVIYRKLGAGLEQVRFTTLRNTVAEASATDTQKYLM
                 ############################################################


                        910       920       930       940       950       960
                 =========+=========+=========+=========+=========+=========+
Ec-11_004410     ALCREEEAKSDPLGNKGHMKSN-GDAVAIGGKHYSGVLRARQLTKLLLNAPLLSLSRLHV
MSTRG.26963.1    ALCREEDAKSDHLGNKGLIKNNKGDAIAIGGKHYSGVLRARQLAKLLLNAPLLSLSRLHV
                 ###################### #####################################


                        970       980       990      1000      1010      1020
                 =========+=========+=========+=========+=========+=========+
Ec-11_004410     TALMSQANILDGMVNYIELVPMIAKTIEGMFHPWSLKMRHEIFNNNRGPGGGGGVGGGMG
MSTRG.26963.1    TALMSQAHIVDGMVNYV-------------------------------------------
                 ################                                            


                       1030      1040      1050      1060      1070      1080
                 =========+=========+=========+=========+=========+=========+
Ec-11_004410     EGGSQQQQGAGTATVRGKSRDDIQRKIVEILREDKQGAAIERLRDPATFARCMASLGLDL
MSTRG.26963.1    ------------------------------------------------------------
                                                                             


                       1090      1100      1110      1120      1130      1140
                 =========+=========+=========+=========+=========+=========+
Ec-11_004410     EPDTINALWVAADLDTREGTTFVDVVDFCFNCVLFMEREKEVKILQRARSTGTSASQSSV
MSTRG.26963.1    ------------------------------------------------------------
                                                                             


                       1150      1160      1170      1180
                 =========+=========+=========+=========+====
Ec-11_004410     PGRAAPGAAAVARAEAVAAAALAAGEPSAGKVDPLLSLARSLVR
MSTRG.26963.1    --------------------------------------------
```

```
Parameters used
Minimum Number Of Sequences For A Conserved Position: 2
Minimum Number Of Sequences For A Flanking Position: 2
Maximum Number Of Contiguous Nonconserved Positions: 8
Minimum Length Of A Block: 10
Allowed Gap Positions: None
Use Similarity Matrices: Yes
```

```
Flank positions of the 3 selected block(s)
Flanks: [623  634]  [679  922]  [924  976]  

New number of positions in Ec-11_004410.aa.fasta.trans.aa_ali.fasta-gb:  309  (26% of the original 1184 positions)
```
